# Inertia–Acoustophoresis Hybrid Microfluidic Device for Rapid and Efficient Cell Separation

**DOI:** 10.3390/s22134709

**Published:** 2022-06-22

**Authors:** Uihwan Kim, Byeolnim Oh, Jiyeon Ahn, Sangwook Lee, Younghak Cho

**Affiliations:** 1 Department of Mechanical Design and Robot Engineering, Seoul National University of Science & Technology, 232 Gongneung-ro, Nowon-gu, Seoul 01811, Korea; them10@naver.com; 2 Department of Electronic Engineering, Kwangwoon University, 20 Kwangwoon-ro, Nowon-gu, Seoul 01897, Korea; o_byeoulnim@naver.com; 3 Institute of Precision Machinery Technology, Seoul National University of Science & Technology, 232 Gongneung-ro, Nowon-gu, Seoul 01811, Korea; 4 Division of Radiation Biomedical Research, Korea Institute of Radiological & Medical Sciences, 75 Nowon-ro, Nowon-gu, Seoul 01812, Korea; ahnjy@kirams.re.kr; 5 Bio-Health Product Research Center, Inje University, 197 Inje-ro, Gimhae-si 50834, Gyeongsangnam-do, Korea; 6 PCL, Inc., Rm 701, Star Valley, 99 Digital-ro 9-gil, Geumcheon-gu, Seoul 08510, Korea; 7 Department of Mechanical System Design Engineering, Seoul National University of Science & Technology, 232 Gongneung-ro, Nowon-gu, Seoul 01811, Korea

**Keywords:** inertial prefocusing, acoustophoresis, serpentine microchannel, particle/cell separation

## Abstract

In this paper, we proposed an integrated microfluidic device that could demonstrate the non-contact, label-free separation of particles and cells through the combination of inertial microfluidics and acoustophoresis. The proposed device integrated two microfluidic chips which were a PDMS channel chip on top of the silicon-based acoustofluidic chip. The PDMS chip worked by prefocusing the particles/cells through inducing the inertial force of the channel structure. The connected acoustofluidic chips separated particles based on their size through an acoustic radiation force. In the serpentine-shaped PDMS chip, particles formed two lines focusing in the channel, and a trifugal-shaped acoustofluidic chip displaced and separated particles, in which larger particles focused on the central channel and smaller ones moved to the side channels. The simultaneous fluidic works allowed high-efficiency particle separation. Using this novel acoustofluidic device with an inertial microchannel, the separation of particles and cells based on their size was presented and analyzed, and the efficiency of the device was shown. The device demonstrated excellent separation performance with a high recovery ratio (up to 96.3%), separation efficiency (up to 99%), and high volume rate (>100 µL/min). Our results showed that integrated devices could be a viable alternative to current cell separation based on their low cost, reduced sample consumption and high throughput capability.

## 1. Introduction

As traditional methods for the separation of micro-particles and blood cells, centrifuge- and membrane-based filters have been widely used. However, the centrifugation method has many disadvantages in that it is time-consuming and expensive, requiring skilled labor and a large sample volume, and the membrane-based filtering method is limited by the membrane pore size and clogging. The need for efficient cell separation, which is an essential preprocessing step in biological and chemical analysis, clinical diagnostics, and environment detection, has led to the recent development of numerous microfluidic separation techniques. Microfluidic separation techniques have distinct advantages, such as fast separation rates, improved accuracies and portability, simple operating procedures, low cost, and a small amount of reagent compared with conventional separation methods, such as centrifuge- and membrane-based filtering [1,2]. 

Generally, microfluidic separation techniques can be classified as active and passive methods, regardless of whether the external force is applied. Active separation methods usually employ an external force field such as acoustic [3], dielectric [4], or magnetic [5] forces for the selective manipulation of different cell groups. They have advantages, such as good separation efficiency, high accuracy, and great control flexibility, but they are expensive because a driving unit is required to drive the device, and the installation and operation of the equipment are complicated. 

On the other hand, passive separation methods have various separation principles, which are based on geometry, such as channel shape and embedded structures, as well as the intrinsic physical properties (size, shape, density, and elasticity) of cells, which includes hydrophoretic [6] and hydrodynamic filtration [7,8], deterministic lateral displacement (DLD) [9], gravitational sedimentation [10], and inertial focusing [11,12]. These passive separation methods have the great advantages of high throughput sample handling, low cost, simple experimental setup, and operation with low energy consumption, but the performance control is not easy.

A hybrid cell separation system combining the advantages of active and passive methods shows great potential to achieve the high-throughput, high-accuracy cell separation of complex and heterogeneous samples [13]. Several studies have recently been published on high-efficiency particle and cell separation using hybrid microfluidics [14,15,16,17,18,19,20,21,22,23,24]. The hybrid systems were generally divided into two regions, which involved a prefocusing region before a separation one. Prefocusing prevents a “field dispersion” that works by limiting the initial position of the particles to a designated place through the process. After prefocusing, the hybrid system can improve its separation efficiency while it performs the separation of particles and cells. The above-mentioned active (acoustophoresis, magnetophoresis, electrophoresis, etc.) and passive (hydrophoresis, deterministic lateral displacement (DLD), inertial focusing, etc.) methods were used as prefocusing methods. 

Laurell et al. used two acoustic systems for prefocusing and particle separation [14,15,16,17,18]. Two PZT transducers were used to separate target particles and cells; one was for performing prefocusing in the front of the device, and the other was for actual separation in the back of the device. This method has advantages such as a simple particle separation process, less damage to the sample, and a label-free technique. However, disadvantages include low throughput, slow processing and low separation efficiency for a sample with a high concentration, and it also requires two acoustic systems.

Yan et al. [19,20] used dielectrophoresis (DEP)-active hydrophoretic devices to sort particles and cells, which consisted of a prefocusing region and a sorting region. It provided a sorting method based not only on size but also on the dielectric properties of the particles or cells of interest without any labeling. Additionally, it was used to extract plasma from diluted whole blood. Chen et al. [21] proposed a hybrid method for microparticle separation based on a delicate combination of induced charge electro-osmosis (ICEO) focusing and dielectrophoretic deflection. Luo et al. [22] presented a simple microfluidic separator for the continuous and label-free separation of cells, which combines gravitational-sedimentation-based sheathless prefocusing and DEP separation methods. However, DEP strongly depends on the conductivity of media, so it is difficult to achieve particle/cell separation in buffers with high conductivity.

Several groups used inertial focusing for the prefocusing of cell sorting and separation. Zhou et al. [23] presented a hybrid microfluidic cell-sorting method combining size-dependent inertial prefocusing and the fluorescence activated acoustic sorting at the single-cell level. They used a reverse wavy channel for differential cell prefocusing due to the size difference, and the prefocused cells were acoustically sorted. Tottori et al. [24] combined the deterministic lateral displacement (DLD) separation method with inertial prefocusing for size-based particle separation. They successfully separated 7 μm and 13 μm particles using sheathless DLD methods after inertial prefocusing along a straight rectangular input channel. However, the DLD method can be only used for a limited particle size. Zhang et al. [25] used inertial focusing for separating leukocytes and erythrocytes by connecting two inertial microfluidic chips. This has several advantages such as a simple device structure, high-throughput and fast processing, little damage to the sample, and continuous separation. However, it is possible only at a high flow rate for particle separation, and it is difficult to integrate into a single chip.

**Figure 1:**
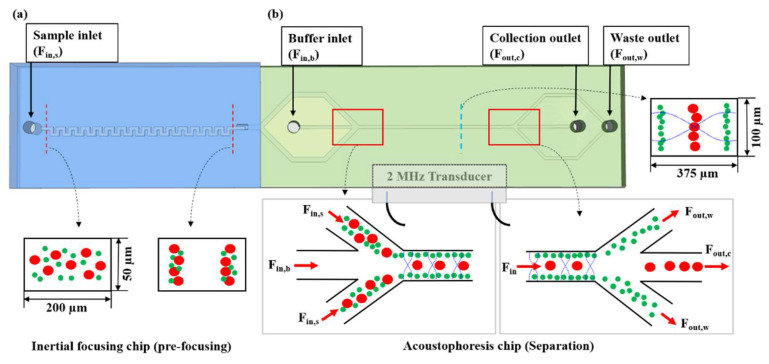
Schematic view of inertia–acoustophoresis hybrid microfluidic device. (**a**) PDMS serpentine-shaped chip for inducing inertial focusing during particle flowing. Two lines of particles were aligned near the wall. (**b**) Acoustophoresis chip for particle separation. Using acoustic radiation force, particles were separated based on their size and collected into each outlet.

In this research, we propose a hybrid microfluidic device, which consists of two microfluidic chips, namely, the serpentine-shaped PDMS chip connected to the trifugal-shaped acoustofluidic chip (see Figure 1). The serpentine-shaped chip works by prefocusing the particles in two rows through induced inertial force, and the acoustofluidic chip separates particles based on size using an acoustic radiation force.

By combining both chips, the hybrid microfluidic device can improve the separation efficiency and recovery ratio with a high volume rate. As the mixture of samples entered the acoustic standing wave field, the larger particles (or cells) migrated across the central buffer interface and exited through the central outlet, whereas the smaller particles (or cells) remained in the original buffer stream along the sidewalls and were removed through the side outlets, as shown in Figure 1. The efficacy of the device was characterized using three parameters, namely, the recovery ratio, separation efficiency, and enrichment factor. The separated targets and non-targets were collected in the target outlet and waste outlet, respectively, via flow control. The recovery ratio and separation efficiency were >96.3% and 99%, with an enrichment factor of 108. Furthermore, we demonstrated an on-chip hybrid microfluidic device for separating a target microalgal species from a mixed population of microalgae in a rapid and continuously running manner. 

As a proof of principle, the device was tested using microalgae cells, since microalgae currently gain a great interest from academic and industry field. Microalgae are morphologically and physiologically diverse unicellular microorganisms found in all marine ecosystems [26]. Providing a potential source of sustained biofuel [27], they convert solar energy and carbon dioxide (CO_2_) into biomass via photosynthesis [28,29], grow rapidly with a typical doubling time of 24 h, and contain high levels of bio-oil [30,31]. The rapid growth rates and capacity to generate large quantities of lipids offer the species a significant advantage as a source of renewable energy [32,33,34]. Moreover, since microalgae can grow in simple and inexpensive conditions, requiring only sunlight, free seawater, inexpensive nitrogen, phosphorus, and carbon, making them ideal candidates for producing commercial bio-products such as recombinant proteins, fine chemicals, pharmaceutics, and feed stocks [35,36,37,38,39].

## 2. Materials and Methods

### 2.1. Design and Fabrication Process of Device

Figure 1 shows the schematic view of the integrated acoustofluidic devices with the inertial microchannel. The entire chip can be divided into three functional regions: inertial prefocusing region, sheath flow region, and acoustophoretic separation region. In this research, we proposed a serpentine microchannel for the prefocusing of particles and cells. Based on [25], we designed and fabricated two serpentine microchannels (Chip 1) in the experiment, which had a 20 mm length and 27 zigzag periods. The depth and width of the microchannels were 50 μm × 200 μm and 100 μm × 400 μm, respectively. The acoustofluidic chip (Chip 2) was designed to allow acoustophoretic separation at ~2 MHz actuation of PZT. The depth and width of the microchannel were 100 μm and 375 μm, and the length of the microchannel affected by acoustic actuation was 30 mm. Additionally, in the inlet part of Chip 2, the depth and width of the microchannel were 100 μm and 550 μm so that the particles coming out of Chip 1 were not affected by acoustic waves. 

**Figure 2:**
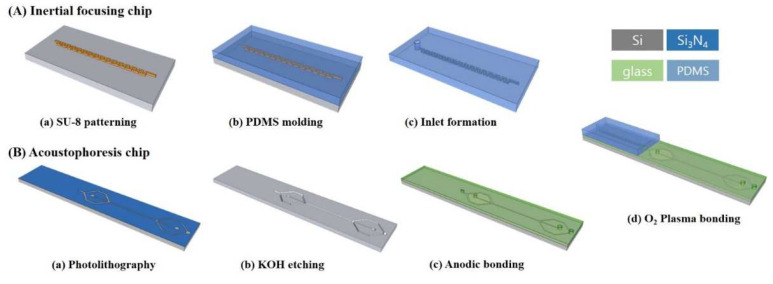
Fabrication process and fabricated device. (**A**) Inertial focusing chip. (**B**) Acoustophoresis chip.

The standard soft lithography technique was employed to fabricate the PDMS inertial microchannel as shown in Figure 2A. Negative photoresist SU-8 50 (Kayaku Advanced Materials, Inc., Westborough, MA, USA) was spin-coated to obtain the final layer thickness of 50 μm and 100 μm. After soft baking at a temperature of 95 °C on a hotplate for 7 min, it was exposed to UV light using a mask aligner (MDA-400M, Midas System Co., LTD., Daejeon, Korea). After post-exposure baking at a temperature of 95 °C on a hotplate for 7 min, the photoresist layer was developed using an SU-8 developer. The fabricated SU-8 master mold was coated with silane (tridecafluoro-1,1,2,2-tetrahydrooctyl trichlorosilane) for the easy release of the PDMS structure from the SU-8 master mold. A PDMS was mixed with a curing agent and poured onto an SU-8 master mold. After degassing in a vacuum chamber for 30 min, it was cured at 80 °C for 1 h. The PDMS microchannel was peeled off and punched with a 2 mm-diameter puncher for the connection of the tube. Finally, the inertial microchannel was bonded onto acoustofluidic chip using O_2_ plasma (Figure 1). 

Figure 2B shows the fabrication process of the acoustofluidic chip (Chip 2) using basic MEMS processes (photolithography, reactive ion etching (RIE), KOH anisotropic etching of Si) and anodic bonding between Si and glass. In brief, a Si_3_N_4_ thin film layer of 1000 Å thickness was deposited on a (100) single crystal Si wafer using low-pressure chemical vapor deposition (LPCVD) and patterned using photolithography and RIE (Figure 2(Ba)). The channel pattern was formed at 45° to the primary flat of the Si wafer to generate the vertical side walls of the microchannel. The Si wafer was anisotropically etched with KOH solution at 70 °C (Figure 2(Bb)). The glass and silicon were anodically bonded at 400 °C by applying a 700 V DC voltage for 40 min (Figure 2(Bc)). After the bonding process, silicone tubing was bonded onto the holes of the glass using PDMS structures for fluidic access to the device.

### 2.2. Reagent and Cell

Fluorescent polystyrene (PS) particles (Thermo SCIENCE Inc., Waltham, MA, USA) with sizes of 5 and 13 μm were dispersed in the DI water, and the surfactant Tween 20 (Sigma-Aldrich, St. Louis, MO, USA) was added to the suspensions at 0.1 wt% to prevent particle aggregation in the particle separation experiments. For the separation test, two kinds of polystyrene particle were mixed to a final concentration of 7 × 10^6^ counts/mL (5 μm) and 2.65 × 10^5^ counts/mL (13 μm) in the DI water, respectively. 

The unicellular green alga, Haematococcus lacustris (20~30 μm) were obtained from the National Institute for Environmental Studies (Tsukuba, Japan). The wild-type of *C. reinhardtii* (CC-125, 2~10 μm) was obtained from the Chlamdomonas Resource Center (CRC, St. Paul, MN, USA).

### 2.3. Experimental

In the inertial prefocusing experiments, the polystyrene particles (microbeads) with similar sizes to relevant microalgae were used to observe the inertial focusing behavior (such as inertial equilibrium positions and distribution) in the serpentine microchannel. The sample flow (F_in,s_) was continuously infused into the Chip 1 using a syringe pump (LEGATO 111, KD Scientific Inc., Holliston, MA, USA) with a controlled volumetric flow rate. It was set to 70~115 μL/min for polystyrene particles with a 50 μm × 200 μm channel and to 300~900 μL/min for microalgae with 100 μm × 400 μm, respectively. The trajectories of fluorescent particles were recorded using a CMOS camera (E3ISPM05000KPA, Touptek Photonics Co., Ltd., Hangzhou, China) on an inverted microscope (BX-60, Olympus, Tokyo, Japan) to capture their inertial focusing behavior. The sheath flow (Dulbecco’s phosphate-buffered saline, DPBS, Thermo SCIENCE Inc., Waltham, MA, USA) from the buffer inlet (F_in,b_) of Chip 2 was also injected using another syringe pump, whose rate was from 50 to 70 μL/min for polystyrene particles with a 50 μm × 200 μm channel and to 270~350 μL/min for microalgae with 100 μm × 400 μm, respectively. 

In the acoustophoretic experiments, the ultrasonic standing waves were induced using a piezoelectric transducer (PZT) (PI41670, PI Ceramic GmbH, Lederhose, Germany), which was attached underneath the acoustofluidic chip with cyanoacrylate instant adhesive (Loctite 401, Henkel AG & Co. KGaA, Lederhose, Germany), and wires were soldered to the PZT for electrical interconnection. It was actuated using function generators (AFG2021, Tektronix Inc., Beaverton, OR, USA) equipped with power amplifier circuits (7058, Power Amplifier, Yokogawa, Japan) to generate a sinusoidal signal, and the voltage over PZT transducer was measured using an oscilloscope (TBS 1104, Tektronix Inc., Beaverton, OR, USA). Since the acoustic half wave matched the size of the channel for 1D lateral separation (See Figure 1b), its resonance frequency was 1.95 MHz whose actuation voltages ranged from zero to 15 V_p-p_. 

All the analyses and postprocessing of the captured images were performed using the open-source ImageJ software. In this work, the fluorescent intensity profile across the channel was extracted and fitted using Gaussian distribution.

## 3. Results and Discussion

### 3.1. Inertial Prefocusing Using Serpentine Microchannel

Particle migration using inertial force under Newtonian fluid in straight channels is induced by the shear gradient lift force (F_LS_) and the wall lift force (F_LW_). The shear gradient lift force F_LS_ arises from the parabolic fluid velocity profile, directing toward the channel wall. On the other hand, the wall lift force (F_LW_) originates from wall-induced disturbance when particles are near the channel wall, always pointing to the channel centerline. The equilibrium position of particle focusing is a result of the balance of two inertial forces. If the particle size is far smaller than the channel size, the net inertial lift force *F_L_* can be expressed as follows [40,41].

*F_L_* = *ρ*_f_ *V_m_*^2^ *a*^4^/*D_h_*^2^
*f*_*L*_(Re, z) (1)

Re = *ρ*_f_ *V_m_ D_h_*/*μ* (2)

where *ρ*_f_, *V_m_*, *a*, *D_h_* and *μ* are the fluid density, maximum velocity, particle diameter, hydraulic diameter of the channel, and dynamic viscosity, respectively. *f_L_* (Re, z) is the dimensionless coefficient of the net inertial lift force, which is a function of the particle position within a cross-section of the channel (z) and channel Reynolds number (Re) [41].

In microfluidic channels with a curvature, a pressure gradient along the radial direction occurs due to the fluid momentum mismatch in the center and near-wall region within the curvature, which subsequently induces a secondary flow perpendicular to the main flow direction; that is, Dean flow will occur in the form of two counter-rotating Dean vortices. Accordingly, it exerts a Dean drag force (*F_D_*) on the particle perpendicular to the main flow, and it is calculated as follows:

(3) $$F_{D}=3\pi \mu aV_{D}\sim \;\frac{\rho V_{m}^{2}aD_{h}^{2}}{R}$$

where *V_D_* is the magnitude of the Dean flow and *R* is the radius of the curvature. 

The additional lateral force (Dean drag force) from secondary flow could affect the inertial focusing and position of particles because the Dean drag force (*F_D_*) is comparable to the inertial lift force (*F_L_*) [42]. As a result, by comparing the balance between the two forces, it is possible to achieve the size-based particle separation in continuous flow due to the size-dependent differential particle focusing, which produces the differential equilibrium positions of varying particle sizes [43].

**Figure 3:**
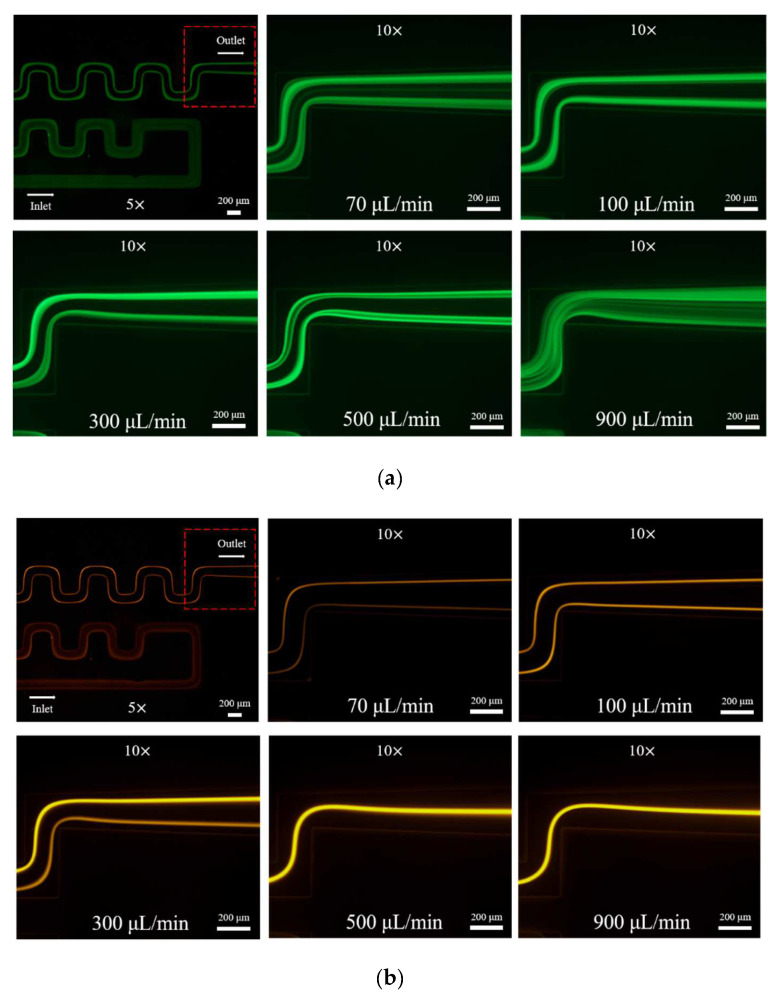
Prefocusing performance of PDMS chip. The fluorescent images of particle trajectory in different zigzag periods of a serpentine channel according to the flow rate from 70 μL/min to 900 μL/min. (**a**) The 5 μm particle, (**b**) The 13 μm particle.

The particle focusing behavior (e.g., inertial equilibrium positions and distribution) in the symmetrical serpentine microchannel (Chip 1) was first investigated. To evaluate the effect of Re on the particle focusing, the 5 μm (green) and 13 μm (red) beads were infused into the device, and the fluorescence intensities of the flowing beads were measured for different sample flow rates (F_in,s_) between 70 μL/min (Re = 9.3) and 900 μL/min (Re = 120) as shown in Figure 3. The infused particles were broadly distributed across the width of the channel near the inlet port and gradually migrated toward their equilibrium positions. All particles were focused to two streams along the sidewalls when increasing the flow rate from 70 to 300 μL/min (Re = 9.3~40). Above a certain Re threshold, however, the large particles (13 μm) migrated towards the channel centerline, and their focusing streams merged into a single beam along the channel centerline. In contrast, small particles (5 μm) still formed two focusing lines along two sidewalls to obtain wider flow rates.

### 3.2. Separation Test of Particles and Cells

The acoustophoresis chip can displace flowing particles or biological cell in the microchannel. Using acoustic radiation force through the actuation of the PZT, the chip enables the transfer of particles from one carrier fluid to another and separates particles based on their sizes or physical properties, such as density and compressibility [15,16]. The vibration generated by PZT activation propagates and forms the resonance pattern and pressure gradient in the microchannel. The particles displace to either pressure minima or maxima, such as the nodal (or antinodal) point, based on their size, density, and compressibility according to Equations (4) and (5).

*F*_rad_ = 4π*a*^3^*ϕ k_y_E_ac_*sin(2*k_y_y*) (4)

where

*ϕ* = (*κ_o_* − *κ_p_*)/3*κ_o_* + (*ρ*_p_ − *ρ*_o_)/(2*ρ*_p_ + *ρ*_o_) (5)

*F*_rad_ denotes the acoustic radiation force applying to the particle which has its radius of *a*, *ϕ*, *k_y_* and *E_ac_* are the acoustic contrast factor, the wave number, and the acoustic energy density, respectively. *y* defines the distance from the wall along the axis of the standing wave. *κ_o_* is the isothermal compressibility of the fluid whereas *κ_p_* is the that of the particle. *ρ_p_* and *ρ_o_* are the density of the particle and the fluid, respectively. The particle velocity induced by acoustic radiation denote as *v*, when balanced by Stokes’ drag in a fluid with viscosity.

(6) $$v=\frac{2\Phi }{3\eta }a^{2}k_{y}E_{ac}sin\left(2k_{y}y\right)$$

In general, the acoustic radiation force becomes stronger against larger and denser particles compare to smaller ones, and its difference in acoustic mobility can transfer the particles into different lateral flow positions (flow stream) [44]. 

Next, we evaluated the particle separation in the integrated acoustofluidic chip, which was located after the particle prefocusing region. For the sample flow rate (F_in,s_) of 100 μL/min, which was chosen because better separation performances were obtained through the repeated experiments in various conditions, both the 5 μm and 13 μm beads were focused near the two sidewalls of the channel, and then entered the separation region of the acoustofluidic chip. The prealigned particles flowing from the inertial focusing chip entered the acoustophoresis chip (See Figure 1), and the particles displaced toward the nodal plane located on the channel center. Due to PZT actuation, particles of different sizes could be separated and collected into different outlets where the larger particles moved to the central outlet of the separation channel (collection outlet (F_out,c_)) and the other particles were collected in the side outlet (waste outlet (F_out,w_)), as shown in Figure 1b.

Mixture microbeads samples (7 × 10^6^ counts/mL (5 μm): 2.65 × 10^5^ counts/mL (13 μm)) from the sample inlet (F_in,s_) were prepared and injected using sheath flow from the buffer inlet (F_in,b_) of the hybrid microfluidic device, while the electrical power was applied on the PZT to drive acoustic radiation force in the acoustofluidic channel, as shown in Figure 1. The particles were focused by inertial focusing in Chip 1, and their prefocusing was maintained even after the particles moved from Chip 1 to Chip 2. A pressure node was formed in the center of the channel by the acoustophoresis in the chip when a frequency of approximately 1.95 MHz was applied. Therefore, the large particles moved to the center of the channel (F_out,c_) due to their sufficient acoustic force, but the small particles did not move to the center of the channel and transferred to the side outlet (F_out,w_). 

Table 1 shows the experimental results of the separation of 5 μm green particles and 13 μm red particles according to the experimental conditions, such as sample flow rate, sheath flow rate and voltage applied to PZT. As the applied voltage increased, the separation efficiency also increased. However, the recovery ratio decreased for 6 V due to the focusing of the small particles to the center. Moreover, the increasing sample flow rate can improve the separation efficiency and recovery ratio.

The recovery ratio, separation efficiency, and enrichment factor were calculated by direct observing of particle (or cell) movement in the separation channel using microscope system including a high-speed camera (PHANTOM VEO-E 310L, Vision Research Inc., Wayne, NJ, USA) with the frame rate over 1200 fps (See Figure 1). They were defined as the follows [16].

(7) $$\mathrm{Recovery}\;\mathrm{ratio}\;=\frac{N_{\mathrm{target}}^{\mathrm{out}1}}{N_{\mathrm{target}}^{\mathrm{in}}}$$

(8) $$\mathrm{Separation}\;\mathrm{efficiency}\;=\frac{N_{\mathrm{target}}^{\mathrm{out}1}}{N_{\mathrm{target}}^{\mathrm{out}1}+{\;N}_{\mathrm{target}}^{\mathrm{out}2}}$$

(9) $$\mathrm{Enrichment}\;\mathrm{factor}\;=(\frac{N_{\mathrm{target}}^{\mathrm{out}1}}{N_{\mathrm{non}-\mathrm{target}}^{\mathrm{out}1}})/(\frac{N_{\mathrm{target}}^{\mathrm{in}}}{N_{\mathrm{non}-\mathrm{target}}^{\mathrm{in}}})$$

**Figure 4:**
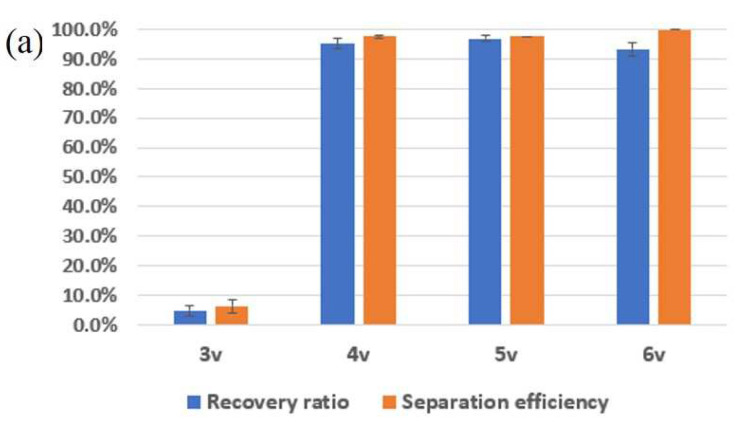

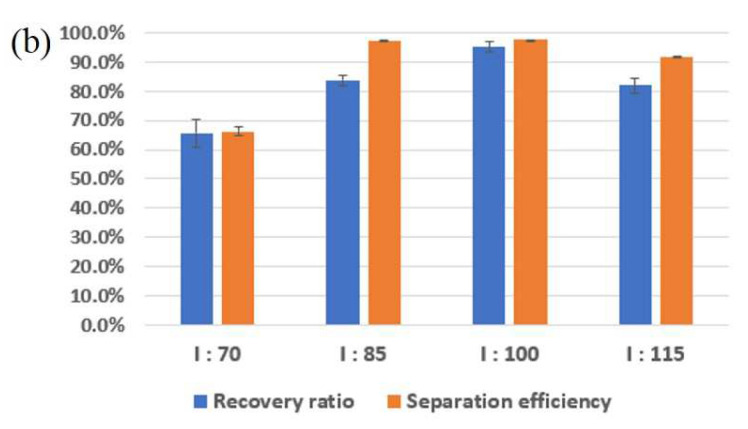
Recovery and separation efficiency from two different size of microbeads. (**a**) Inertial flow rate (F_in,s_): 100 μL/min; sheath flow rate (F_in,b_): 70 μL/min (fixed); x-axis: applied voltage (varied); (**b**) sheath flow rate: 70 μL/min; applied voltage: 4 V (fixed); x-axis: inertial flow rate (varied).

Figure 4a shows the recovery ratio and separation efficiency of the particles in the various voltage conditions at the sample flow rate of 100 μL/min and sheath flow rate of 70 μL/min, respectively. At 4 V, the recovery ratio and separation efficiency were >92% and >95%. Figure 4b represents the recovery ratio and separation efficiency whilst varying the sample flow from 70 μL/min to 115 μL/min at a fixed applied voltage of 4 V. The recovery ratio improved from 65% to 99%, while the separation efficiency changed from 67% to 96.3%, as shown in Figure 4b. Additionally, the maximum enrichment factor almost reached 108. On the other hand, the recovery ratio (95% from 96.3%) and separation efficiency (97% from 99%) decreased a little without the serpentine channel for prefocusing in the same conditions. However, the enrichment factor dramatically decreased from 108 to 23. This means that the hybrid microfluidic channel with prefocusing channel works better for the enrichment of targets as well as for the separation and recovery of targets (Supplementary Video S1).

**Figure 5:**
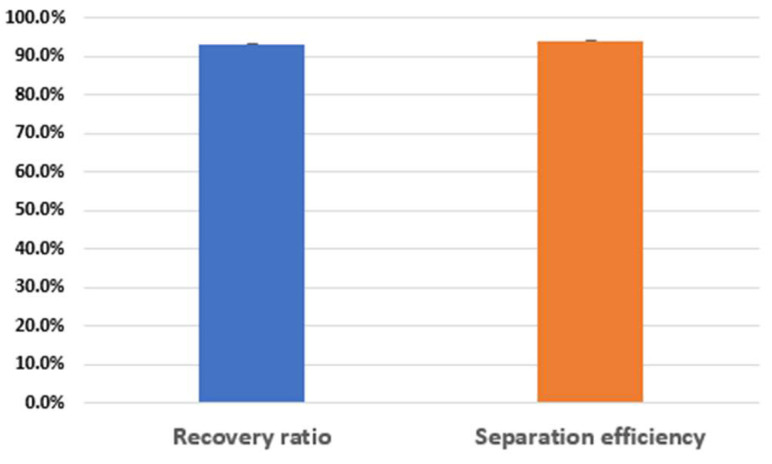
Recovery and separation efficiency from Chlamydomonas reinhardi (CC-125) and Haemotoccocus lacustris. Inertial flow rate (F_in,s_): 300 μL/min, sheath flow rate (F_in,b_): 300 μL/min, applied voltage: 14 V.

As a proof of principle of separation performance, we selected two kinds of size different microalgae cell named *Chlamydomonas reinhardtii* (CC-125, 2~10 μm, 1.41 × 10^6^ cells/mL) and *Haemotoccocus lacustris* (20~30 μm, 1.35 × 10^5^ cells/mL), and tested the separation experiments for the mixture sample of the microalgae cells (Supplementary Video S2). The prepared sample was injected into the inlet port of the hybrid microfluidic device, while electrical power was applied to the PZT to drive acoustic radiation force in the acoustofluidic channel, as shown in Figure 1. For the applied voltage of 14 V, sample flow rate of 300 μL/min and sheath flow rate of 300 μL/min, large *Haemotoccocus lacustris* moved to the center outlet (F_out,c_), while small CC-125 moved out to the combined side outlet (waste outlet (F_out,w_)). The experimental results showed that the recovery ratio and separation efficiency were >93.2% and >94%, with an enrichment factor of 261, as shown in Figure 5. It means that the proposed device works properly in separating cells as well as microbeads.

## 4. Conclusions

In this study, a three-dimensionally integrated microfluidic device for particle/cell separation using inertial prefocusing and acoustophoresis was proposed. It consisted of passive inertial microchannels (Chip 1) and an acoustophoresis chip (Chip 2), which were fabricated using the basic MEMS process. The two chips were three-dimensionally integrated and connected, followed by O_2_ plasma bonding, to construct the final integrated device. The particles were pre-focused in two rows by inertial force after passing through the inertial focusing microchannel, and then large and dense particles moved to the center line of the channel at a higher speed than small and less dense particles via acoustophoretic force. Using the fabricated device, particles and microalgae cells were successfully separated according to their size and density, and their separation efficiency and recovery ratio were presented and analyzed. Compared to well-known separation methods such as centrifugation or filtration, the proposed acoustophoretic method including inertial prefocusing can demonstrate the separation of microalgae cells in a continuous and biocompatible manner, which can overcome the limitation of efficiency and biocompatibility. In the future, the proposed device will be optimized through further experiments for use as a powerful tool for the preparation of biological samples such as those of circulating tumor and blood cells.

## Figures and Tables

**Table 1:** Fluorescent microscope images of green (5 μm) and red (13 μm) polystyrene particles at the bifurcation of outlet for various experimental conditions such as sample flow rate, sheath flow rate and applied voltage.

Sample Flow Rate (μL/min)	Sheath Flow Rate (μL/min)	Applied Voltage (V)	5 (μm)	13 (μm)
70	50	4	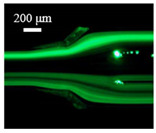	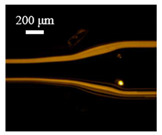
		5	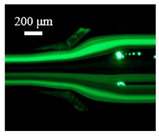	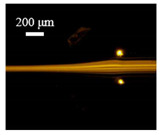
100	50	3	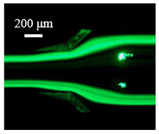	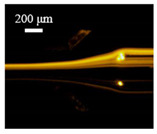
	70	3	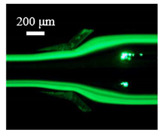	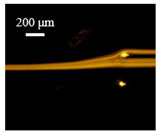
		4	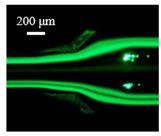	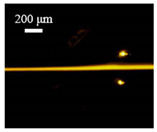
115	70	4	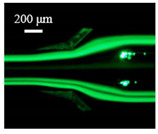	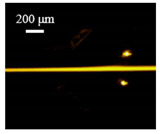
		5	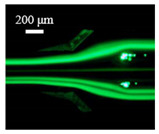	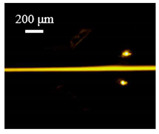

## Data Availability

Not applicable.

## Supplementary Materials

The following supporting information can be downloaded at: https://www.mdpi.com/article/10.3390/s22134709/s1, Video S1. Separation of 5 μm (two sides) and 13 μm polystyrene particles (center) at the bifurcation of outlet for sample flow rate of 100 μL/min, sheath flow rate of 70 μL/min and applied voltage of 4 V without prefocusing (no use of the serpentine channel); Video S2. Separation of *Chlamydomonas reinhardtii* (CC-125, 2~10 μm) (two sides) and *Haemotoccocus lacustris* (20~30 μm) (center) at the bifurcation of outlet for sample flow rate of 300 μL/min, sheath flow rate of 300 μL/min and applied voltage of 14 V with prefocusing.
